# The Prevalence of Attention Deficit/Hyperactivity Disorder Symptoms in Children and Adolescents With Autism Spectrum Disorder Without Intellectual Disability: A Systematic Review

**DOI:** 10.1177/10870547231177466

**Published:** 2023-06-07

**Authors:** Christopher Eaton, Kayley Roarty, Nimisha Doval, Smitarani Shetty, Karen Goodall, Sinead M. Rhodes

**Affiliations:** 1 University of Edinburgh, UK; 2 Cardiff University School of Medicine, UK; 3 Neurodevelopment Service for Children and Young People, Newmains Health Centre, Lanarkshire, UK; 4 Child and Adolescent Mental Health Services, NHS Grampian, UK; 5 Child and Adolescent Mental Health Services, NHS Lothian, UK

**Keywords:** autism spectrum disorder, attention deficit hyperactivity disorder, prevalence, co-occurrence

## Abstract

**Objective:**

ADHD commonly co-occurs with ASD without ID in young people. It has been difficult to obtain accurate prevalence estimates of ADHD in this population, as a dual-diagnosis was not permitted until DSM-V. We systematically reviewed the literature on the prevalence of ADHD symptoms in young people with ASD without ID.

**Method:**

9,050 articles were identified through six databases. Articles were reviewed against inclusion and exclusion criteria and 23 studies were included.

**Results:**

ADHD symptom prevalence varied from 2.6% to 95.5%. We discuss these findings according to the ADHD assessment measure, informant, diagnostic criteria, risk of bias rating and recruitment pool.

**Conclusion:**

ADHD symptoms are common in young people with ASD without ID, but there is substantial variance in study reporting. Future studies should recruit participants from community sources, provide information on key sociodemographic sample characteristics and assess ADHD with standardized diagnostic criteria, using both parent/carer and teacher report.

## Introduction

Autism spectrum disorder (ASD) is a heterogeneous neurodevelopmental disorder with a global prevalence estimate of 1% (Lyall et al., 2017). ASD is characterized by persistent difficulties with social communication, social interaction and repetitive and restrictive behaviors (American Psychiatric Association, 2013; World Health Organization [WHO], 2004). It has been estimated that ~70% of children with ASD experience co-occurring psychiatric disorders (Abdallah et al., 2011; American Psychiatric Association, 2013). Attention Deficit Hyperactivity Disorder (ADHD), is defined by pervasive symptoms of inattention, hyperactivity and impulsivity, which are functionally impairing across home and school settings (American Psychiatric Association, 2013; Mazzone et al., 2012). ADHD has been commonly reported in ASD (Leitner, 2014). Co-occurrence of ADHD and ASD in children is unsurprising, considering the overlaps in age of onset, behavioral problems, and difficulty in social skills (Craig et al., 2015; Joshi et al., 2017).

The Diagnostic and Statistical Manual of Mental Disorders only allowed for a dual-diagnosis in its fifth edition (American Psychiatric Association, 2013). Prior to this, there was much controversy around a co-occurring diagnosis, with many researchers describing ADHD symptoms as “just a part of autism” (Mansour et al., 2017). Following the publication of the DSM-V, the landscape of research around the co-occurrence of these conditions changed and allowed for better clinical management and a clearer understanding of the overlap of these disorders (Leitner, 2014).

Diagnostic constraints have limited the evidence on the impact of co-occurring ADHD and ASD, where studies employing DSM-IV criteria have often excluded individuals with co-occurring psychiatric conditions (Davis & Kollins, 2012). It has however been established that the presence of ADHD exacerbates the severity of impairments in children with ASD (Sprenger et al., 2013; Yamawaki et al., 2020). Children with ASD and co-occurring ADHD show greater social and cognitive impairments, higher rates of internalizing and externalizing behaviors (Holtmann et al., 2007; Rao & Landa, 2014; Visser et al., 2016) and conduct problems (Jang et al., 2013). Identifying ADHD symptoms in children with ASD is therefore important for interventions which can achieve optimal social and behavioral outcomes during a child’s critical stages of development (Belmonte et al., 2004; Dawson, 2008; Srinath & Jacob, 2016).

Neuropsychological difficulties shared by ASD and ADHD have been reported as similar due to comparable genetic loads and endophenotypes, despite a lack of overlap in the diagnostic criteria of both disorders (Ghirardi et al., 2019; Jang et al., 2013). Rommelse et al. (2011) suggested ADHD and ASD share similar endophenotypes, including difficulties with emotion regulation, social awareness, and externalizing behaviors. This overlap may account for the severity of impairment present in children with a co-occurring diagnosis.

Literature and systematic review studies have attempted to estimate the prevalence of co-occurring ADHD and ASD, but are limited by the inclusion of adults and those with ID. Lai et al. (2019) meta-analyzed co-occurring psychiatric symptoms in adults and children with ASD, across a range of IQ and observed an ADHD prevalence of 22% in community samples, increasing to 34% in clinical samples. Reviewing the co-occurrence of ASD and ADHD in children with mixed IQ levels, Leitner (2014) reported estimates ranging from 37% to 85% in clinic samples, noting that rates were expected to be lower in community samples of varying ages. Rong et al. (2021) meta-analyzed the current and lifetime prevalence of ADHD in adults and children with ASD, including those with intellectual disability. Current prevalence estimates in the 6 to 11 and 12 to 17 age groups were both 47.8%, whereas lifetime prevalence estimates were 39.4% and 38.8%, respectively. A meta-regression, including the whole sample, revealed that studies with more participants with ID were associated with significantly lower current prevalence estimates. This review did not however provide the figures for the prevalence of ADHD in autistic people with and without ID.

To date there have been no reviews providing estimates of ADHD prevalence in young people with ASD without ID specifically. It is important to separate out this group when estimating ADHD prevalence, because evidence suggests low IQ may be a potential confound when assessing ADHD prevalence in young people with ASD. Witwer and Lecavalier (2010) found that the profile of ADHD symptoms in autistic young people with ID was different for those without ID; overall fewer ADHD symptoms were endorsed for those with ID, with higher rates specifically for “push their way into groups” and “interrupts others.”

This review examines the prevalence of ADHD symptoms in autistic children and adolescents without ID. Given that a dual-diagnosis of ADHD and ASD was not permitted until DSM-V, we include studies which focused on the prevalence of ADHD symptoms, alongside those where participants meet criteria for a clinical diagnosis of ADHD.

Our review addresses the limitations of previous studies estimating the prevalence of ADHD in ASD in a number of ways. We are the first review to provide estimates of the prevalence of ADHD in young people with ASD without ID specifically. Second, the review compares estimates of ADHD prevalence provided by (i) parents or caregivers (ii) teachers, (iii) both (mixed). This breakdown of prevalence by type of informant was not included in the reviews conducted by Lai et al. (2019) and Rong et al. (2021). Lai et al. (2019) only included estimates of ADHD prevalence based on parent report. Rong et al. (2021) included prevalence estimates based on teacher report, but where a study had provided separate estimates based on both parent and teacher report they chose to include the parent-report estimate in their meta-analysis. This is important because research into the prevalence of ADHD-symptoms has consistently found that parent reports tend to lead to higher estimates than teacher reports (Narad et al., 2015; Wolraich et al., 2004), suggesting a strong parent report bias.

Specifically, this review aimed to:

1. Identify the prevalence of ADHD symptoms, as well as a clinical diagnosis of ADHD, in young people with ASD without ID aged 5 to 19 years.
2. Appraise the methodological quality of included studies, including the measures used to assess ADHD symptoms in this population.
3. Make recommendations for future studies looking to assess the prevalence of ADHD in this population.

## Methodology

### Reporting

This review was conducted and written in accordance with the Preferred Reporting Items for Systematic Reviews and Meta Analyzes (PRISMA) Checklist (Moher et al., 2009). The protocol for this review was registered on PROSPERO (CRD42020182156).

### Search Strategy

The search strategy was defined by identifying four key terms from the research question: “autism,” “ADHD” “prevalence” and “child/adolescents.” Common synonyms and Medical subject headings (MESH) for these terms were extracted from previous reviews on the prevalence of ADHD (Polanczyk et al., 2007; Thomas et al., 2015) and ASD (Wigham et al., 2017).

The four search lines below were combined with the Boolean operator “AND”:

1. autis* OR asperger* OR “ASD” OR “ASC” OR “high functioning ” OR “HFA” OR “pervasive developmental disorder*” OR “PDD”
2. “Attention Deficit Hyperactivity Disorder” OR “ADHD” OR “attention deficit disorder” OR “ADD” OR “hyperkinetic syndrome” OR “hyperkinetic disorder” OR “attention deficit” OR “attention disorder” OR hyperactiv* OR inattent* OR impulsiv*
3. “prevalence” OR “epidemiology” OR “rate” OR “frequency”
4. child* OR adoles* OR youth* OR minor* OR girl* OR boy* OR teen* OR pediatr* OR paediatr* OR “young person”

These terms were searched in six databases: Cinahl, EMBASE, ERIC, MEDLINE, PsychINFO and Web of Science, and limited to studies conducted after 1992, when the WHO defined Asperger’s Syndrome (World Health Organisation, 1992). The search was conducted on 7th May 2020 and updated on 23rd October 2021. Results were limited to those studies involving human participants, published in the English language within peer-reviewed journals. Duplicate records were removed from the results.

### Selection, Inclusion and Exclusion

Table 1 displays the inclusion and exclusion criteria. Studies with young people aged 5 to 19 were included, in which ASD was clinically diagnosed according to DSM-IV, DSM-V, or ICD-10 criteria, or where a diagnosis was given using a validated assessment tool, such as the Autism Diagnostic Observation Schedule (ADOS; (Lord et al., 2000) or the Autism Diagnostic Interview, Revised (ADI-R; (Lord et al., 1994). Included studies were required to assess the triad of ADHD symptoms (inattentiveness, hyperactivity, and impulsiveness), and present these data for the ASD group without ID specifically. ASD without ID was defined as participants with a full-scale IQ (FSIQ) ≥70.

**Table 1.** Inclusion and Exclusion Criteria.

Inclusion	Exclusion
Data for young people aged 5 to 19 years was available or the mean age was ≥5 years and ≤ 19 years	Data for young people ≥5 years and ≤ 19 years could not be separated from preschool children or adults
ASD was diagnosed according to DSM-IV, DSM-V, or ICD-10 criteria; or on a validated diagnostic tool (ADOS or ADI-R)	ASD was diagnosed prior to the DSM-IV or ICD-10; or a screening tool was used; or diagnostic procedure was not reported
ASD without ID was assessed as FSIQ ≥ 70	Studies recruiting children with ID did not provided a separable ASD without ID (FSIQ ≥ 70) group; or FSIQ assessment was not reported
The prevalence of ADHD symptoms was available from the ASD without ID group	ADHD symptoms were reported as a mean score; or the number of participants meeting ADHD cut-off scores was not provided
For intervention studies, the prevalence of ADHD symptoms was available pre-intervention	Pre-intervention data on the prevalence of ADHD symptoms was not available
For longitudinal studies, baseline or follow-up data on the prevalence of ADHD symptoms was available	Longitudinal baseline or follow-up ADHD assessment data was not available

*Note*. ASD = autism spectrum disorder; ADHD = attention deficit/hyperactivity disorder; ID = intellectual disability; ADOS = Autism Diagnostic Observation Schedule; ADI-R = Autism Diagnostic Interview, Revised.

**Figure 1.**
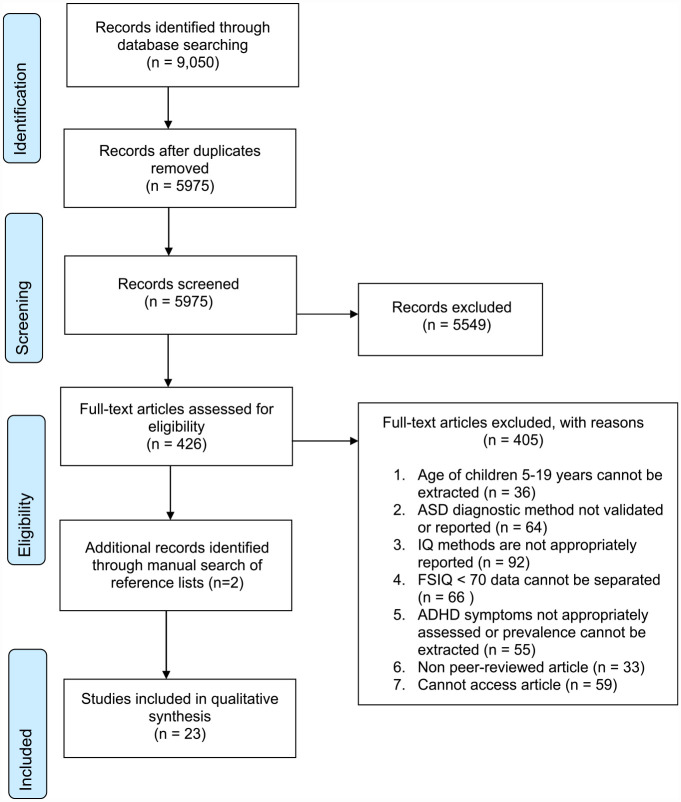
PRISMA flow diagram.

Titles and abstracts of articles obtained from the database search were screened against the inclusion and exclusion criteria (original search K.R., updated search N.D.), with 20% being independently reviewed by a second reviewer (C.E.). Agreement was 96%. Articles which met the inclusion criteria at the screening stage were then reviewed in full (original search K.R., updated search N.D.) and 20% were independently reviewed (C.E.). Agreement was 91%. All discrepancies at each stage were resolved until 100% consensus was met. A senior reviewer was consulted if final decisions remained unclear (S.R.). In order to identify additional relevant articles not captured by the database search, backward citation searching was performed using the reference lists of articles which met the inclusion criteria at full-text review. Full details of the selection process are provided in Figure 1.

### Data Extraction and Quality Assessment

All data were independently extracted by two reviewers (K.R. and C.E.). Disagreements were resolved until 100% consensus was made. Missing information was recorded as not reported (NR). Where available, data were extracted for children with ASD without ID unless stated otherwise. The data extracted was: (1) country of study, (2) study type (cross-sectional/longitudinal) (3) number of participants with ASD without ID, (4) recruitment pool, (5) mean age, standard deviation (SD) and range, (6) percentage of male participants, (7) ethnicity, (8) socio-economic status, (9) medication status, (10) co-occurring conditions, (11) diagnostic criteria utilized for ASD assessment, (12) who provided ASD diagnosis, (13) specific ASD diagnosis given, (14) additional ASD research diagnosis measures, (15) when the participant's IQ was assessed, (16) full scale intelligence quotient (FSIQ) mean, SD and range, (17) measure used to assess FSIQ, (18) diagnostic criteria for ADHD diagnosis, (19) who provided ADHD diagnosis, (20) tool used to assess ADHD symptoms, (21) who provided information on ADHD diagnosis, (22) psychometric properties reported in the ADHD tool for ASD without ID sample, and (23) the prevalence of ADHD symptoms in the ASD without ID group. The prevalence rate was extracted by assessing the number of children with ASD who displayed co-occurring ADHD symptoms, divided by the total number of children with ASD, expressed as a percentage.

The quality of included studies was independently assessed by two reviewers (K.R. & C.E.), according to validated frameworks which addressed questions on prevalence (Hoy et al., 2012; Munn et al., 2014), and the prevalence of depression in children with ASD without ID (Wigham et al., 2017). Studies were scored as either low (8–10), medium (4–7), or high (0–3) risk of bias. No study was excluded on the basis of a poor quality rating. The individual scales were: (1) Diagnosis of ASD, (2) Assessment of ADHD Symptoms, (3) Clear Description of Participants, (4) Description of Recruitment Pool, and (5) Measure of IQ. Please see the Supplemental Materials for the quality appraisal tool used in this review, with details of adaptations made.

### Data Analysis

The included studies differed in sample size, age range and diagnostic criteria when classifying ADHD symptoms. Studies also used very different assessment tools to measure ADHD. These ranged from questionnaires designed to screen for ADHD specifically (e.g., the ADHD rating scale) to more general psychiatric interview tools (e.g., the Kiddie Schedule for Affective Disorders and Schizophrenia), to unspecified clinical interviews and DSM checklists, reviewed by psychiatrists. In addition, none of the included studies were rated as low risk of bias across all quality assessment scales (see Table 3) and the majority were rated as medium risk of bias in their assessment of ADHD symptoms. It was therefore not possible to meta-analyze ADHD prevalence estimates. The presence of bias in included studies may lead to erroneous or misleading findings when summarized within a meta-analysis (Higgens & Green, 2011). Prevalence estimates of ADHD symptoms across studies are therefore discussed narratively.

## Results

### Search Results

Firstly, 9,050 articles were identified from searching all databases. A total of 3,075 duplicates were removed, leaving 5,975 articles. At title and abstract screening, 5,549 articles were removed. The full texts of 426 articles were read and 21 met the inclusion criteria. An additional two articles were identified from background citation searching, yielding a total of 23 articles included in this review.

### Description of Included Studies

Characteristics of included studies are displayed in Table 2. Across studies, sample sizes ranged from 8 (Ogino et al., 2005) to 1838 participants (Hanson et al., 2013). Samples were predominantly male; the study with lowest percentage of males was 50% (Ogino et al., 2005). The age of participants ranged from 4 (Hanson et al., 2013) to 51 years (Ghaziuddin et al., 1998). Whilst this range exceeds the reviews age criteria (5–19 years), both studies reported a mean sample age ≥5 years and ≤ 19 years and therefore were retained. The medication status of participants was reported by 10 studies (Adamo et al., 2014; Andersen et al., 2013; Biscaldi et al., 2016;

**Table 2.** Characteristics of Included Studies.

Study	ASD without ID n (% male)	Age range (M, SD)	FSIQ range (M, SD); measure	SES	Ethnicity	Recruitment Pool (country)	ASD assessment	ADHD assessment	Prevalence of ADHD: n/N (%)
Adamo et al. (2014)	46 (91%)	7–11.9 years (*M* = 10, *SD* = 1)	Range NR, FSIQ > 80 (*M* = 109, *SD* = 17), WASI	Range 4 or 5 (high class)-71% (32/46), Index of Social Position (Hollingshead, 1957)	NR	Community: study centers, family associations, parent support groups & advertisements (Germany)	DSM-IV-TR criteria. Diagnosis of AD, AS and PDD-NOS by clinicians and based on child’s history combined with ADOS module 3 and ADI-R	DSM-IV-TR criteria, omissions of criterion E. Diagnosis based on K-SADS-PL interviews with parent only (*n* = 18), parent-child dyads (n = 11), or unstructured psychiatric interviews with parents (*n* = 17), administered by clinicians. Symptoms: CPRS-R:L, completed by parents (cut off T-score ≥ 65)	CPRS-L: 23/46 (50%) K-SADS-PL : 17/46 (37%)
Andersen et al. (2013)	38 (81.6%)	9–17 years (*M* = 12, *SD* = 2.3)	Range NR, FSIQ > 70, (*M* = 98.2 *SD* = 17.8), WASI	NR	NR	Clinical: Child and Adolescent Mental Health Centers (Norway)	DSM-IV criteria. K-SADS-PL reviewed by a senior clinician, supplemented by ASSQ Diagnoses of AS & PDD-NOS	CBCL completed by parents (cut off T-score ≥ 65)	16/38 (42.1%)
Biscaldi et al. (2016)	28 (82%)	Range NR (*M* = 10.7, *SD* = 1.8)	Range NR, FSIQ > 70, (*M* = 108.7, *SD* = 21), SPM	NR	NR	Clinical: Department of Child and Adolescent Psychiatry & Psychotherapy, University of Freiburg (Germany)	DSM-IV and ICD-10 criteria, supplemented by ADOS and ADI-R. Diagnosis of child autism or AS	DSM-IV criteria. K-SADS-PL administered to parents and children. ADHD was additionally tested by results of the DISYPS (FBB-ADHD), the CBCL, and verified by expert clinicians	10/28 (35.7%)
Caamaño et al. (2013)	25 (96%)	7–17 years, (*M* = 12.8, *SD* = 2.9)	Range NR, FSIQ > 85, (*M* = 97.9, *SD* = 27.6), WISC	NR	23 Caucasians, 2 “other races”	Mixed: Spanish Asperger syndrome family association and clinic, Universitario Gregorio Maranon (Spain)	DSM-IV-TR and Gillberg criteria, supplemented by developmental history and ADOS-G, administered by psychiatrists. Diagnosis of ASD in all participants	DSM-IV-TR criteria, supported by K-SADS-PL, administered to parents and children	17/25 (68%)
Cremone-Caira et al. (2019)	101(86.1%)	7–11 years, (*M* = 9.1, *SD* = 1.4)	Range NR, FSIQ > 70, (*M* = 106.1, *SD* = 14.4), WASI-2	NR	NR	Community: participant registries (U.S.)	Existing diagnosis of ASD supplemented by ADI-R and ADOS-2	CBCL completed by caregiver (cut-off T-score ≥ 65)	39/101 (38.6%)
Duncan et al. (2019)	51 (90.2%)	13–18 years (*M* = 16.3, *SD* = 1.3)	Range NR, FSIQ > 70, (*M* = 98.4, *SD* = 16.5), SB5	17.6% US <$50,000, 31% US $51,000-$100,000, 25.5% $101,000-$160,000, 11.8% US $161,000, 13.7% Declined to answer	86.2% white, 7.9% biracial, 3.9% black, 2.0% Asian, 0% Hispanic	Clinical: outpatients from existing study (Country NR)	Participants with ASD. ADOS-2 administered by researcher	CBCL ADHD subscale completed by caregiver (cut off T-score ≥ 70)	8/51 (15.7%)
Ghaziuddin et al. (1998)	20 (35 in total, however 15 were adults) (80.6% from full sample)	8–51 years, (*M* = 15.1, *SD* = 10.5)	Range NR, FSIQ > 70, (*M* = 102.7, *SD* = 18.7), WISC-R	NR	NR	Clinical: medical referrals (U.S.)	DSM-IV and ICD-10 criteria. All participants diagnosed with AS	DSM-IV criteria. Diagnosis based on psychiatric records obtained from school and social services, psychiatric examination, clinical interviews, checklists and chart reviews. Secondary assessments used K-SADS-E for 8 children aged < 17 years	10/20 (50%)
Gurkan et al. (2008)	11 (90.9%)	7–17 years (Mean = 11.6 years, *SD* = 3.4 years)	Range = 72–126 (*M* = 90.4, *SD* = 16.46 ) WISC-R	NR	NR	Clinical: Autism Diagnosis, Treatment and Research Center, Ankara Univeristy (Turkey)	DSM-IV-TR criteria, supplemented by ASSQ and ASDIF	DSM- IV criteria K-SADS-PL administered by clinician, conducted with parent and child. Presence of PDD not taken as exclusion criteria	5/11 (45.5%)
Hanson et al. (2013)	1838 (89.1%)	4–18 years, (*M* = 8.9, *SD* = 3.5)	Range = 70–167 (*M* = 96.24, *SD* = 16.36), DAS-II, MSEL;, WASI, WISC-IV	NR	62.4% white, 4.0% mixed race, 1.8% African American, 1.5% Asian, 0.1% Native American, 07% other, 39.6% not specified	Clinical: existing study (U.S.)	DSM-IV-TR criteria, supported by ADOS and ADI-R. Participants diagnosed with ASD or PDD-NOS	CBCL (caregiver reports) and TRF for (teacher reports, cut off T-score ≥ 70 on both measures)	CBCL: 324/1838 (17.6%) TRF: 68/813 (8.3%) CBCL & TRF (2.6%)
Kusaka et al. (2014)	49 (73.5%)	6–15 years, (*M* = 11.2, *SD* = 2.5)	Range NR, FSIQ > 70, (*M* = 95.7, *SD* = 13.1), WISC-III	NR	NR	Clinical: outpatient clinic in Osaka City University Hospital (Japan)	DSM-IV-TR criteria: diagnosed with AD, & AS by child psychiatrists. Buitelaar and Van Der Gaag (1998) criteria used to diagnose PDD-NOS.	DSM-IV-TR criteria. K-SADS-PL—parent report. Presence of PDD not taken as exclusion criteria	24/49 (49%)
Mattila et al. (2010)	50 (76%)	9–16 years, (*M* = 12.7, *SD* = 1.5)	Range NR, FSIQ > 75 (M NR, SD NR) WISC-III	NR	NR	Mixed: existing studies (Finland)	DSM-IV-TR criteria; consensus diagnoses of AS and autism without ID from pediatrician, psychiatrist and psychologist. Based on ADI-R, ADOS, patient records, school day observations and ASSQ	DSM-IV criteria supported through K-SADS-PL. Parents and children interviewed by psychiatrist and educational psychologist	Current: 19/50 (38%) Lifetime: 22/50 (44%)
Mazefsky et al. (2011)	38 (82%)	10–17 years, (*M* = 12, *SD* = 2)	Range = 71–144 (*M* = 105, *SD* = 17), WASI	NR	Caucasian 89.5%, African-American 2.6%, Hispanic 2.6%, Bi-racial 5.3%	Mixed: word of mouth and children’s hospital (NR)	ADOS and ADI-R to diagnose AD, AS & PDD-NOS, confirmed by clinical psychologist.	DSM-IV-TR criteria. Diagnosis based on the ACI-PL, administered by first author with parents and children	Subthreshold 22/38 (57.9%) Full DSM-IV-TR 14/38 (36.8%)
Mazefsky et al. (2014)	37^a^ (91.4%)	7–19 years, (*M* = 14.0, *SD* = 2.6)	Range NR, FSIQ > 80, (*M* = 108.2, *SD* = 11.5), WASI	NR	NR	Community: advertisements; presentations to parents and professionals (NR)	Used autism cut-offs in ADI-R and ADOS-G	CBCL completed by parents (cut-off T score ≥65)	10/37 (27%)
Mukaddes et al. (2010)	60 (100%)	Autism without ID group: 6.2 to 14.4 years, median age: 10.3 AS group; 7 -15.5 years, median age = 11.0	Autism without ID group: range = 70 to 127 (median = 90.5, *SD* = NR) AS group: range = 82 to 138 (median = 106.5, *SD* = NR) WISC-R	NR	NR	Clinical: Autism Clinic of Child and Adolescent Psychiatry Department, Istanbul University (Turkey)	DSM-IV criteria. Consensus diagnoses of AS and autism without ID from two psychiatrists, supplemented by parent interview	DSM-IV criteria. K-SADS-PL, administered by two psychiatrists. Presence of autism not taken as exclusion criteria	39/60 (65%)
Mukaddes and Fateh (2010)	37 (86.5)	6–20 years (*M* = 10.9, *SD* = 4.5)	Range = 76–141 (M NR, SD NR) WISC-R	NR	NR	Clinical: referral from 2002 to 2007 at private psychiatry clinic (Turkey)	DSM-IV criteria. AS diagnosed by interviewing child and parents	DSM-IV criteria. K-SADS-PL with parents and children. Developmental history taken from parents	17/37 (45%)
Ogino et al. (2005)	8 (50%)	Group II: 6–12 years, (*M* = 9, SD NR)	Group II: range = 80–105 (*M* = 93.7, *SD* = NR) WISC-III	NR	NR	Clinical: Okayama University Hospital (Japan)	DSM-IV criteria, omission of criterion E: diagnoses for AS from interviews with parents; child daily home activities and school achievements	DSM-IV criteria assessed through interviews with parents and observations of children. Presence of PDD not taken as exclusion criteria	6/8 (75%)
Rau et al. (2020)	419 (76%)	6–18 years, (*M* = 11.1, *SD* = 3.5)	Range = 70–143 (*M* = 99.2, *SD* = 17.1) WASI-II, WAIS-IV, WISC-IV, DAS-II	NR	Data available for 317 participants: 65.93% Caucasian, 13.88% African American, 6.31% Asian/Pacific Islander, 7.57% mixed/other, 27.96% Hispanic	Clinical: private neuropsychiatric rehabilitation and medical center (Finland)	DSM-V criteria, supplemented by ADOS or ADOS-2 and ADI-R by clinician. All participants diagnosed with ASD	DSM-V criteria assessed through interviews by clinicians. ADHD-RS-IV & ASEBA, CBCL and TRF; parent and teacher report. Neuropsychological assessments	259/419 (61.8%)
Reinvall et al. (2016)	60 (80%)	6.5–16.7 years (*M* = 11.6, *SD* = 2.5)	Range NR, FSIQ > 70 (*M* = 105.5, *SD* = 14.5) WISC-III	Maternal education: 6.7% lower, 43.3% medium, 50% higher. Paternal education: 11.7% lower, 45% medium, 40% higher^b^	NR	Project database and recruitment from clinical settings (Finland)	ICD- 10 criteria, supplemented by ADI- R—experienced clinicians and multidisciplinary teams. All participants diagnosed with AS	DSM- IV and ICD 10 criteria assessed through DAWBA completed with parents by clinician	16/60 (26.7%)
Rosa et al. (2016)	50 (92%)	7–17 years, (*M* = 12.0, *SD* = 3.1)	Range NR, FSIQ > 70, (*M* = 101.2, *SD* = 13.8), WAIS-III and WISC	*M* = 45.56, *SD* = 16.81, Index of Social Position (Hollingshead, 1957)	82% Caucasian	Clinical: recruited from Department of Child and Adolescent Psychiatry and Psychology of Hospital Clinic in Barcelona (Spain)	DSM-IV-TR criteria. ASD diagnosis confirmed by ADI-R, administered by trained psychologist.	DSM-IV criteria assessed through K-SADS-PL, administered by trained psychologist	23/50 (46%)
Witwer and Lecavalier (2010)	22 (82%)^c^	6–17 years (*M* = 11.2, *SD* = 3.8)	Range 42–150 (Mean = 68.4, *SD* = 23.3) SB5	1.8% attended high schools, 10.9% graduated high school, 34.5% attended college, 49.1% graduated college, 3.6% graduate/professional school	77% Caucasian	Mixed: Ohio State University-based clinics, local psychiatric offices and support groups, previous OSU psychiatric research participants) (U.S.)	Existing ASD diagnosis confirmed through ADI-R with parent, administered by trained researcher	DSM-IV criteria. Diagnosis based on P-ChIPS conducted with parent, administered by trained researcher	ADHD any subtype: 21/22 (95.5) ADHD combined subtype: 16/22 (72.7%) ADHD inattentive subtype 5/22 (22.7%) ADHD hyperactive subtype: 0/22 (0%)
Yerys et al. (2019)	*Caregiver– report*: 347 (87.3%) *Teacher—report*; 153 (90.8%)	*Caregiver group*: 6–17 years, (*M* = 10.4, *SD* = 2.9) *Teacher group*: 6–17 years, (*M* = 10, *SD* = 3)	FSIQ > 70 *Caregive*r *group*: range NR (M GCA = 100.5, *SD* = 18). *Teacher group*: range NR (M GCA = 99.6, *SD* = 17.4) DAS-II	NR	NR	Mixed: specialty clinics and local advertising (U.S.)	DSM-IV-TR criteria. Diagnosed with ASD by “expert clinical judgment” and informed by ADI-R and ADOS	ADHD-RS-IV, completed by parents and teachers	Caregiver report: 179/347 (51.6%) Teacher report: 108/153 (70.6%)
Yoshida and Uchiyama (2004)	53(90.6%)	7–15 years, (*M* = 10.3, SD NR)	Range NR, FSIQ > 70, (*M* = 87, *SD* = 20), WISC-III	NR	NR	Clinical: outpatients (Japan)	DSM-IV criteria. Diagnosed with AD, AS and PDD-NOS.	DSM-IV criteria. ADHD-RS-IV, parent and teacher report. ADHD symptoms observed by a child psychiatrist and psychologist. Interviews conducted with caregivers	36/53 (67.9%)
Zajic et al. (2018)	77 (81.8%)	8–16 years, (*M* = 11.3, *SD* = 2.1)	Range NR, FSIQ > 70 HF ASD-H group (*M* = 98.0, *SD* = 14.8) WASI—II	Mother’s Education: 2% completed high school, 66% completed college, 33% completed graduate school. Father’s Education: 8% completed high school, 66% completed college, 25% completed graduate school.	6% Asian; 65% Caucasian; 8% Caucasian plus other ethnicity; 10% Hispanic/Latino; 10% Other, 1% Decline to state	Community: university research project, schools and word of mouth (NR)	Existing diagnosis of ASD confirmed by ADOS, administered by a trained researcher and by SCQ, ASSQ and SRS.	Conner’s -3, completed by parents (cut off T-score >69)	52/77 (67.5%)

*Note*. ASD = autism spectrum disorder; ID = intellectual disability; *M* = mean; *SD* = standard deviation; FSIQ = full scale intelligence quotient; SES = socioeconomic status; ADHD = attention deficit/hyperactivity Disorder; WASI = Wechsler Abbreviated Scale of Intelligence (Weschler, 1999); DSM-IV = Diagnostic and Statistical Manual of Mental Disorders, Fourth Edition (American Psychiatric Association, 1994); DSM-IV-TR = Diagnostic and Statistical Manual of Mental Disorders, Fourth Edition, Text Revision (American Psychiatric Association, 2000); DSM-V = Diagnostic and Statistical Manual of Mental Disorders, Fifth Edition; AD = autistic disorder; AS = Asperger’s syndrome; PDD-NOS = pervasive developmental disorder—not otherwise specified; Conners *R* = Conners Parent Rating Scale—Revised: Long Version; NR = not reported; ICD-10 = International Classification of Diseases, 10th Edition (World Health Organisation, 1992); DISYPS (FBB-ADHD) =Diagnostik-System für Psychische Störungen nach ICD-10 und DSM-IV für Kinder und Jugendliche; K-SADS-E = Kiddie Schedule for Affective Disorders and Schizophrenia, Epidemiological Version; ACI-PL = Autism Comorbidity Interview, Present and Lifetime Version; SB5: Standard-Binet Intelligence Scales, Fifth Edition (Roid, 2003); SPM = Standard Progressive Matrices (Raven & Horn, 2009); WISC = The Wechsler Intelligence Scale for Children (Wechsler & Kodama, 1949); WASI-II = Wechsler Abbreviated Scale of Intelligence (Wechsler, 2011); WISC-R = The Wechsler Intelligence Scale for Children, Revised; WAIS-III = The Wechsler Adult Intelligence Scale, Third Edition (Wechsler, 2001); WAIS-IV = The Wechsler Adult Intelligence Scale, Fourth Edition (Wechsler, 2008); DAS-II = Differential Abilities Scales, Second Edition (Elliott et al., 2007); WISC-IV = The Wechsler Intelligence Scale for Children, fourth edition (Wechsler, 2003); WISC-III = The Wechsler Intelligence Scale for Children, third Edition (Wechsler, 1991); GCA = General Conceptual Ability; ADOS-G = Autism Diagnostic Observation Schedule, Generic; ADOS-2 = Autism Diagnostic Observation Schedule, Second Edition (Lord et al., 2012); ADI-R = Autism Diagnostic Interview, Revised; ASSQ = Autism Spectrum Screening Questionnaire (Ehlers et al., 1999); K-SADS-PL = Kiddie Schedule for Affective Disorders and Schizophrenia, Present and Lifetime; P-ChIPS = Children’s Interview for Psychiatric Syndromes, Parent Version; ADHD-RS-IV = ADHD Rating Scale, Fourth Edition (DuPaul et al., 1997); ASEBA = Achenbach System of Empirically Based Assessment; CBCL = Child Behavior Checklist; TRF = Teacher Report Form; DAWBA = Development and Well-Being Assessment; SCQ = Social Communication Questionnaire (Rutter et al., 2003); SRS = Social Responsiveness Scale (Constantino et al., 2003); MSEL = Mullen Scales of Early Learning (Mullen, 2005).

a In C. A. Mazefsky et al. (2014), demographics and ADHD prevalence data were only extracted for the group without gastrointestinal problems.

b Data on paternal education missing for two participants in Reinvall et al. (2016).

c Percentage male, age, IQ, ethnicity and SES provided for whole sample in Witwer and Lecavalier (2010) (*n* = 61).

Cremone-Caira et al., 2019; Gurkan et al., 2008; Mazefsky et al., 2011; Rosa et al., 2016; Witwer & Lecavalier, 2010; Yerys et al., 2019; Yoshida & Uchiyama, 2004); these included stimulant medication, anti-psychotics and antidepressants. All studies reported FSIQ ≥ 70 on either all or a subsample of their participants, using a validated measure of IQ. Three studies reported a FSIQ ≥ 80 (Adamo et al., 2014; Mazefsky et al., 2014; Ogino et al., 2005) and one reported FSIQ ≥ 85 (Caamaño et al., 2013). Socio-economic status (SES) was reported by six studies, this was parent education or occupation in Adamo et al. (2014), Reinvall et al. (2016), Rosa et al. (2016), Zajic et al. (2018) and Witwer and Lecavalier (2010); and parent income in Duncan et al. (2019). Ethnicity was reported by eight studies (Caamaño et al., 2013; Duncan et al., 2019; Hanson et al., 2013; Mazefsky et al., 2011; Rau et al., 2020; Rosa et al., 2016; Witwer & Lecavalier, 2010; Zajic et al., 2018). The majority of studies were cross sectional, with only two utilizing longitudinal designs (Ghaziuddin et al., 1998; Mukaddes & Fateh, 2010). Six studies were conducted in the United States (U.S.) (Cremone-Caira et al., 2019; Ghaziuddin et al., 1998; Hanson et al., 2013; Rau et al., 2020; Witwer & Lecavalier, 2010; Yerys et al., 2019), three in Japan (Kusaka et al., 2014; Ogino et al., 2005; Yoshida & Uchiyama, 2004), three in Turkey (Gurkan et al., 2008; Mukaddes & Fateh, 2010; Mukaddes et al., 2010) two in Finland (Mattila et al., 2010; Reinvall et al., 2016), two in Germany (Adamo et al., 2014; Biscaldi et al., 2016), two in Spain (Caamaño et al., 2013; Rosa et al., 2016) and one in Norway (Andersen et al., 2013). Four studies did not report any country of study (Duncan et al., 2019; Mazefsky et al., 2011, 2014; Zajic et al., 2018).

### Quality Appraisal

The results of the risk of bias assessment are displayed in Table 3. In total, eight studies were rated low risk of bias (Adamo et al., 2014; Caamaño et al., 2013; Rau et al., 2020; Reinvall et al., 2016; Rosa et al., 2016; Witwer & Lecavalier, 2010; Yoshida & Uchiyama, 2004; Zajic et al., 2018) and the remaining 15 studies as medium risk. No study was scored as having a high risk of bias overall. The majority of studies were rated as low risk in the diagnosis of ASD domain; these comprised studies in which a clinician gave a diagnosis according to DSM or ICD criteria and used a validated research tool to diagnose ASD (e.g., the ADOS and/or ADI-R). One study additionally used Gillberg Criteria (Gillberg & Gillberg, 1989), and where this conflicted with DSM-IV-TR criteria (35% of the sample), the ADOS was administered (Caamaño et al., 2013). A medium risk of bias was applied to six studies in the assessment of ASD diagnosis. These studies only administered the ADOS and/or ADI-R to confirm a research diagnosis (Cremone-Caira et al., 2019; Duncan et al., 2019; Mazefsky et al., 2011, 2014; Witwer & Lecavalier, 2010; Zajic et al., 2018). The majority of studies were rated as medium risk of bias in their assessment of ADHD symptoms, where a questionnaire (e.g., the Conner’s Parent Rating Scale) was administered or assessment was based on either parent or teacher report (and not both). Four studies were deemed as low risk, providing a clinical diagnosis of ADHD by a clinician according to DSM-IV, DSM-V, or ICD-10 criteria across home and school settings (Ghaziuddin et al., 1998; Rau et al., 2020; Reinvall et al., 2016; Yoshida & Uchiyama, 2004). The majority of studies were rated as high risk of bias for the background information they provided about participants; only reporting age and gender. Six studies were rated as medium risk, as they reported an additional characteristic such as ethnicity or SES (Adamo et al., 2014; Caamaño et al., 2013; Hanson et al., 2013; Mazefsky et al., 2011; Rau et al., 2020; Reinvall et al., 2016). The remaining four studies were scored as low risk and provided all key characteristics (Duncan et al., 2019; Rosa et al., 2016; Witwer & Lecavalier, 2010; Zajic et al., 2018). Eleven studies were rated as low risk of bias in how they described their recruitment pool, reporting both the method of referral and setting (Adamo et al., 2014; Caamaño et al., 2013; Gurkan et al., 2008; Mazefsky et al., 2011, 2014; Rau et al., 2020; Rosa et al., 2016; Witwer & Lecavalier, 2010; Yerys et al., 2019; Yoshida & Uchiyama, 2004; Zajic et al., 2018). The remaining studies were rated as medium risk, as they only reported one of these descriptors. The vast majority of studies were rated as low risk of bias in how they assessed FSIQ. These studies used various validated IQ measures, with the majority administering versions of the Wechsler Intelligence Scale for Children (WISC; (Wechsler, 1974). The exception is Rau et al. (2020), rated as medium risk, as the measure used to assess IQ was not reported.

**Table 3.** Quality Appraisal.

Study	Diagnosis of ASD	Assessment of ADHD Symptoms	Description of Participants	Description of Recruitment Pool	Measure of FSIQ	Total Score
Adamo et al. (2014)	Low	Medium	Medium	Low	Low	Low
Andersen et al. (2013)	Low	Medium	High	Medium	Low	Medium
Biscaldi et al. (2016)	Low	Medium	High	Medium	Low	Medium
Caamaño et al. (2013)	Low	Medium	Medium	Low	Low	Low
Cremone-Caira et al. (2019)	Medium	Medium	High	Medium	Low	Medium
Duncan et al. (2019)	Medium	Medium	Low	Medium	Low	Medium
Ghaziuddin et al. (1998)	Low	Low	High	Medium	Low	Medium
Gurkan et al. (2008)	Low	Medium	High	Low	Low	Medium
Hanson et al. (2013)	Low	Medium	Medium	Medium	Low	Medium
Kusaka et al. (2014)	Low	Medium	High	Medium	Low	Medium
Mattila et al. (2010)	Low	Medium	High	Medium	Low	Medium
Mazefsky et al. (2011)	Medium	Medium	Medium	Low	Low	Medium
Mazefsky et al. (2014)	Medium	Medium	High	Low	Low	Medium
Mukaddes et al. (2010)	Low	Medium	High	Medium	Low	Medium
Mukaddes and Fateh (2010)	Low	Medium	High	Medium	Low	Medium
Ogino et al. (2005)	Low	Medium	High	Medium	Low	Medium
Rau et al. (2020)	Low	Low	Medium	Low	Medium	Low
Reinvall et al. (2016)	Low	Low	Medium	Medium	Low	Low
Rosa et al. (2016)	Low	Medium	Low	Low	Low	Low
Witwer and Lecavalier (2010)	Medium	Medium	Low	Low	Low	Low
Yerys et al. (2019)	Low	Medium	High	Low	Low	Medium
Yoshida and Uchiyama (2004)	Low	Low	High	Low	Low	Low
Zajic et al. (2018)	Medium	Medium	Low	Low	Low	Low

Note. ASD = autism spectrum disorder; ADHD = attention deficit/hyperactivity disorder; FSIQ = full scale IQ.

## Assessment of ADHD Symptoms

In total, 16 studies administered interviews. Of these, 10 used the Kiddie Schedule for Affective Disorders and Schizophrenia, Present and Lifetime (K-SADS-PL; (Kaufman et al., 1997)) including German (Biscaldi et al., 2016), Turkish (Gurkan et al., 2008; Mukaddes & Fateh, 2010; Mukaddes et al., 2010), Japanese (Kusaka et al., 2014), and Spanish (Rosa et al., 2016) translations. The K-SADS Epidemiological Version (K-SADS-E; (Puig-Antich et al., 1980) was used to assess children under the age of 17 years in Ghaziuddin et al. (1998). The Autism Comorbidity Interview, Present and Lifetime Version (ACI-PL, Lainhart et al. (2003) was administered by C. Mazefsky et al. (2011). The Finnish version of the Development and Wellbeing Assessment (DAWBA, R. R. Goodman et al. (2000) was used in Reinvall et al. (2016). The parent version of the Children’s Interview for Psychiatric Symptoms (P-ChIPS; (Weller et al., 1999) was used in Witwer and Lecavalier (2010). Several studies employing interviews did not provide details of the tool used: Ogino et al. (2005), Rau et al. (2020), Yoshida and Uchiyama (2004) and Adamo et al. (2014), for 37% of their sample. Interviews were conducted alongside child observations (Ogino et al., 2005; Rau et al., 2020), neuropsychological assessments (Rau et al., 2020), and DSM checklists and reviews (Ghaziuddin et al., 1998). Three studies integrated interview and questionnaire data when estimating ADHD prevalence: Rau et al. (2020) used clinical interviews, the ADHD Rating Scale (ADHD-RS, DuPaul (1991) and the Achenbach System of Empirically Based Assessment (Achenbach, 2001), which included the Child Behavior Checklist (CBCL) and the Teacher Report Form. Yoshida and Uchiyama (2004) combined data from the ADHD-RS and clinical interviews with parents. Biscaldi et al. (2016) used both the K-SADS-PL, the CBCL and the DISYPS FBB-ADHS (Diagnostik-System für Psychische Störungen nach ICD-10 und DSM-IV für Kinder und Jugendliche; Dopfner et al., 2008). One study used the K-SADS-PL and the Conners’ Parent Rating Scale—Revised: Long Version (Conners, 1998) to produce separate prevalence estimates (Adamo et al., 2014).

A total of 11 studies administered questionnaires. Seven assessed ADHD symptoms using the CBCL (Achenbach & Ruffle, 2000); these were Andersen et al. (2013), Biscaldi et al. (2016), Cremone-Caira et al. (2019), Duncan et al. (2019), Hanson et al. (2013), Mazefsky et al. (2014) and Rau et al. (2020), with Hanson et al. (2013) and Rau et al. (2020) additionally administering the Teacher Report Form. The remaining studies used the Conners’ Parent Rating Scale (CPRS, Conners (2008) (Adamo et al., 2014; Zajic et al., 2018); and the ADHD-RS (Rau et al., 2020; Yerys et al., 2019; Yoshida & Uchiyama, 2004).

No study reported on the psychometric properties of measures used to assess ADHD symptoms in young people with ASD without intellectual disability.

### The Prevalence of ADHD Symptoms in ASD Without ID

The prevalence of ADHD symptoms ranged from 2.6% to 95.5% (Hanson et al., 2013; Witwer & Lecavalier, 2010). Prevalence estimates are presented according the measure used to assess ADHD, the type of informant, the criteria used for studies which diagnosed ADHD, the overall risk of bias rating and participant recruitment method.

#### Prevalence by Measure Used to Assess ADHD

The prevalence of ADHD symptoms in studies using interviews ranged from 26.7% to 95.5% (Reinvall et al., 2016; Witwer & Lecavalier, 2010). Studies using versions of the K-SADS-PL reported a prevalence range from 35.7% to 68% (Biscaldi et al., 2016; Caamaño et al., 2013) . The ACI-PL showed a prevalence of 36.8% meeting full DSM-IV-TR criteria and a subthreshold prevalence of 57.9% (Mazefsky et al., 2011). The Finnish version of the DAWBA showed a prevalence of 26.7% (Reinvall et al., 2016). The P-ChIPS showed an overall prevalence of 95.5%, with 72.7% and 22.7% of the sample meeting criteria for the combined and inattentive subtypes, respectively (Witwer & Lecavalier, 2010). The prevalence of studies administering questionnaires was 2.6% to 70.6% (Hanson et al., 2013; Yerys et al., 2019). Studies using the CBCL estimated a prevalence ranging from 2.6% to 61.8% (Hanson et al., 2013; Rau et al., 2020). When looking at studies which used the CBCL to provide a separate prevalence estimate, the prevalence ranged from 2.6% (Hanson et al., 2013) to 42.1% (Duncan et al., 2019). Studies using the CPRS reported prevalence rates of 50% (Adamo et al., 2014) and 67.5% (Zajic et al., 2018). Importantly, cut off scores varied from 65 (Adamo et al., 2014) to 69 (Zajic et al., 2018). The ADHD-RS showed a prevalence of 51.6% to 70.6%, depending on the informant in Yerys et al. (2019). For Yoshida and Uchiyama (2004) and Rau et al. (2020), in which the ADHD-RS was integrated with interview data, the prevalence was 67.9% and 61.9%, respectively.

#### Prevalence by Type of Informant

Eleven studies provided prevalence estimates based on separate reports from parent or caregivers (Adamo et al., 2014; Andersen et al., 2013; Cremone-Caira et al., 2019; Duncan et al., 2019; Kusaka et al., 2014; Mazefsky et al., 2014; Ogino et al., 2005; Reinvall et al., 2016; Witwer & Lecavalier, 2010; Yerys et al., 2019; Zajic et al., 2018). The prevalence of ADHD symptoms in these studies ranged from 15.7% to 95.5%. Seven studies included mixed reports from the young person and parent/caregiver (Adamo et al., 2014; Biscaldi et al., 2016; Caamaño et al., 2013; Gurkan et al., 2008; Mattila et al., 2010; Mazefsky et al., 2011; Mukaddes & Fateh, 2010), with prevalence estimates ranging from 35.7-68%.

Teacher reports were explicitly mentioned by four studies. Yerys et al. (2019) provided a separate estimate based on teacher report of 70.6%. The remaining three studies used mixed teacher and parent reports and observed prevalence rates of 67.9% (Yoshida & Uchiyama, 2004) 61.8% (Rau et al., 2020) and 2.6% (Hanson et al., 2013). Ghaziuddin et al. (1998) included information from schools when assessing comorbidity in their sample, but did not provide further details. The informant was not reported in Mukaddes et al. (2010) and Rosa et al. (2016).

#### Prevalence by Diagnostic Criteria Used

Of the 11 studies applying DSM-IV criteria, prevalence ranged from 26.7% tο 95.5% (Biscaldi et al., 2016; Ghaziuddin et al., 1998; Gurkan et al., 2008; Mattila et al., 2010; Mukaddes & Fateh, 2010; Mukaddes et al., 2010; Ogino et al., 2005; Reinvall et al., 2016; Rosa et al., 2016; Witwer & Lecavalier, 2010; Yoshida & Uchiyama, 2004). DSM-IV-TR criteria was administered by four studies, with a prevalence range from 36.8% to 68% (Adamo et al., 2014; Caamaño et al., 2013; Kusaka et al., 2014; Mazefsky et al., 2011). One study applied DSM-V criteria, reporting a prevalence of 61.8% (Rau et al., 2020).

#### Prevalence by Risk of Bias Rating

For studies rated as low risk of bias, prevalence estimates ranged from 26.7% to 95.5% (Reinvall et al., 2016; Witwer & Lecavalier, 2010), although the majority of these were 50% or above. For those studies rated as medium risk of bias, prevalence estimates ranged from 2.6% to 75% (Hanson et al., 2013; Ogino et al., 2005), although the majority of these were less than 50%.

#### Prevalence by Sample Type (Clinical, Community or Mixed)

Fourteen studies recruited from a clinical sample, with prevalence ranging from 2.6% to 75% (Andersen et al., 2013; Biscaldi et al., 2016; Duncan et al., 2019; Ghaziuddin et al., 1998; Gurkan et al., 2008; Hanson et al., 2013; Kusaka et al., 2014; Mukaddes & Fateh, 2010; Mukaddes et al., 2010; Ogino et al., 2005; Rau et al., 2020; Reinvall et al., 2016; Rosa et al., 2016; Yoshida & Uchiyama, 2004). Community samples were used within four studies, where prevalence ranged from 27% to 67.5% (Adamo et al., 2014; Cremone-Caira et al., 2019; Mazefsky et al., 2014; Zajic et al., 2018). Mixed recruitment methods were used by five studies, with prevalence ranging from 36.8% to 95.5% (Caamaño et al., 2013; Mattila et al., 2010; Mazefsky et al., 2011; Witwer & Lecavalier, 2010; Yerys et al., 2019).

## Discussion

This review examined the prevalence of ADHD symptoms in children and adolescents aged 5 to 19 years with ASD without ID. Prevalence estimates ranged from 2.6% to 95.5% and varied according to salient study characteristics, including the assessment tool, the informant, the risk of bias rating, the study recruitment pool, and the diagnostic criteria used. Even when considering those studies rated as low risk of bias (and therefore of high methodological quality), prevalence estimates still varied widely, from 26.7% to 95.5%. Across studies, there was a dearth of consistent reporting of variables which may feasibly affect prevalence estimates such as SES and ethnicity. It is clear however that the co-occurrence of ADHD is common in autistic young people without ID.

Prevalence estimates were generally higher in studies administering interviews. Whilst some studies administering questionnaires reported some of the highest prevalence estimates for example, 70.6% (Yerys et al., 2019) and 67.5% (Zajic et al., 2018), the majority reported prevalence estimates of less than 50%. Interviews are arguably a more thorough method of asking about ADHD symptoms, due to, for example, the clinical expertise of the interviewer, as well as the opportunity for the respondent to clarify questions and avoid misunderstandings. Questionnaire studies may be underestimating the prevalence of ADHD in young people with ASD without ID. It is important to note however that our findings are only descriptive; Rong et al. (2021) found no significant differences when comparing ADHD prevalence estimates (both current and lifetime) by method of assessment (e.g., clinical interview vs. questionnaire). Studies in this review did not assess or report on the psychometric properties of measures used to assess ADHD in young people with ASD without ID. It is important that tools used to assess ADHD prevalence are shown to be valid and reliable in this population specifically, to ensure accurate prevalence estimates.

The importance of using multiple informants has been emphasized when assessing ADHD symptoms (Martel et al., 2015) and is part of the DSM-V diagnostic criteria for ADHD (American Psychiatric Association, 2013). Despite this, only four studies included teacher informants. Yerys et al. (2019) used teacher reports and observed one of the highest prevalence rates (70.6%), whereas Hanson et al. (2013) found that their prevalence estimate dropped from 17.6% (parent-only report) to 2.6% when including teacher reports. As previously mentioned, in the general population estimates of ADHD symptom prevalence are higher when using parent report compared to teacher reports (Narad et al., 2015; Wolraich et al., 2004), suggesting a strong parent report bias. A lack of studies including teacher reports did not allow for meaningful comparisons to be made in this review. Future studies should endeavor to assess ADHD using both parent and teacher reports, to allow for more accurate prevalence estimates.

The majority of studies rated as low risk of bias reported prevalence estimates of 50% and above. The majority of medium risk studies reported prevalence estimates of less than 50%. In this review, ADHD assessment was considered to be gold-standard if a clinical diagnosis was made using standardized diagnostic criteria (e.g., DSM, ICD), using reports from both parents and teachers. The detail of how ADHD was assessed varied across studies. It is therefore possible that some studies were given a higher risk of bias rating than if detailed information about ADHD assessment been provided. Future studies should provide clear details including the ADHD assessment measure, the informant and whether a diagnosis was given, the criteria used and who gave the diagnosis (e.g., psychiatrist).

The majority of studies recruited participants either exclusively, or partially, from clinical sources. Referral patterns into services can introduce bias when using clinical samples to estimate prevalence (e.g., a threshold level of severity in order to be referred). Research into the prevalence of mental health conditions in young people has shown that clinical samples are likely to be more impaired and have higher levels of co-occurrences than community samples (S. H. Goodman et al., 1997). In addition, the majority of studies in this review had a sample size of less than 100, which may affect the accuracy of prevalence estimates. Future studies should aim to recruit large samples of young people with ASD without ID, drawn from community sources.

Several confounding variables such as SES, ethnicity, geographic region, and gender should be considered when comparing prevalence rates of ADHD symptoms in young people with ASD without ID. All studies reported information regarding the age and gender of their sample, however ethnicity, SES, and geographic region were inconsistently reported. Country of study and ethnicity are particularly important, considering that clinical practice varies largely between cultures (Caron et al., 2012; Norbury & Sparks, 2013). Within this review, a majority of studies conducted within the U.S. reported a prevalence of 50% rate or above, while most European studies reported estimates below 50%. European clinicians have reportedly been more reluctant to diagnosis ADHD in children when compared to U.S. counterparts (Malacrida, 2004). Ethnicity is also important to consider, as lower rates of ADHD are reported in ethnic minority children than in white children (Morgan et al., 2013; Schneider & Eisenberg, 2006). Additionally, low SES has been associated with a higher risk of ADHD in children and adolescents (A. E. Russell et al., 2016). This review was unable to examine differences in prevalence estimates across ethnicity and SES, due to a lack of reporting of these data. In almost all studies included in this review, the majority of participants were male, limiting the generalizability of our findings. This is similar to the findings of Rong et al. (2021), who observed that the proportion of males in most studies included in their meta-analysis was more than 70%. Future studies should endeavor to include more females with ASD without ID, given the increased rate of diagnosis of ASD in females seen in recent years (G. Russell et al., 2022). Studies should also clearly describe other key sociodemographic characteristics of participants, such as ethnicity and SES.

This review has several limitations. Firstly, we made several adaptions to the risk of bias assessment tool; developed from the tools used in Hoy et al. (2012), Munn et al. (2014) and Wigham et al. (2017). These included the removal of criteria assessing the psychometric properties of ADHD assessment tools in children with autism without intellectual disability, as no study provided data on this. Adaptions may have compromised the validity and reliability of the tool. This review only considered children and adolescents of school age (5–19 years). ADHD symptoms often present in children prior to the age of 4 years (Harvey et al., 2009; Turygin et al., 2013). A better understanding of the prevalence of ADHD symptoms in pre-school children with ASD could inform early intervention.

This is the first review to examine the prevalence of ADHD symptoms in children and adolescents with ASD without ID. This review shows that whilst the co-occurrence of ADHD symptoms in this population is common, prevalence estimates vary considerably. We highlight the heterogeneous nature of methodology across studies in this area, (e.g., participant recruitment, the measure used to assess ADHD, the informant), the lack of clear reporting of salient characteristics such as SES and ethnicity, and the lack of data on psychometric properties of ADHD assessment tools when used with autistic young people without ID. To ensure more accurate prevalence estimates, future studies should endeavor to recruit large samples from community sources and to diagnose ADHD using standardized diagnostic criteria, using information from both parents and teachers. It is also important that key sociodemographic characteristics about the sample are accurately reported, as these may be important when considering the generalizability of prevalence estimates for ADHD in children and adolescents with ASD without ID.

## Supplemental Material

sj-docx-1-jad-10.1177_10870547231177466 – Supplemental material for The Prevalence of Attention Deficit/Hyperactivity Disorder Symptoms in Children and Adolescents With Autism Spectrum Disorder Without Intellectual Disability: A Systematic Review Click here for additional data file. Supplemental material, sj-docx-1-jad-10.1177_10870547231177466 for The Prevalence of Attention Deficit/Hyperactivity Disorder Symptoms in Children and Adolescents With Autism Spectrum Disorder Without Intellectual Disability: A Systematic Review by Christopher Eaton, Kayley Roarty, Nimisha Doval, Smitarani Shetty, Karen Goodall and Sinead M. Rhodes in Journal of Attention Disorders
