# Supplementary material for: The Prevalence of Attention Deficit/Hyperactivity Disorder Symptoms in Children and Adolescents With Autism Spectrum Disorder Without Intellectual Disability: A Systematic Review
Source: J Atten Disord. 2023 Jun 7;27(12):1360–76. doi: 10.1177/10870547231177466 (PMC10498659; doi:10.1177/10870547231177466)
Supplement: sj-docx-1-jad-10.1177_10870547231177466 – Supplemental material for The Prevalence of Attention Deficit/Hyperactivity Disorder Symptoms in Children and Adolescents With Autism Spectrum Disorder Without Intellectual Disability: A Systematic Review [file sj-docx-1-jad-10.1177_10870547231177466.docx]

**Quality appraisal tool used in this review**

The quality of included studies was assessed using a tool adapted from validated frameworks which addressed questions on prevalence (Hoy et al., 2012; Munn, Moola, Riitano, & Lisy, 2014), and the prevalence of depression in children with ASD without ID (Wigham, Barton, Parr, & Rodgers, 2017). The individual scales were: (1) Diagnosis of ASD, (2) Assessment of ADHD Symptoms, (3) Clear Description of Participants, (4) Description of Recruitment Pool, and (5) Measure of IQ.

Within each of the below subscales, studies were given a score of 0, 1 or 2 (exception is ‘Measure of IQ’, which is a two-level subscale).

- 0 = High risk of bias
- 1 = Medium risk of bias
- 2 = Low risk of bias

Studies were then given an overall risk of bias score, obtained from totalling the scores from the individual subscales. The total score was either low (8-10), medium (4-7), or high (0-3) risk of bias.

We adapted the aforementioned frameworks for use in this review. These included general changes in order to adapt the frameworks for this specific review topic, for example, specifying that a low risk of bias score should be assigned to those studies which incorporated both parent and teacher report when assessing ADHD symptoms. We also made changes to the tool based on the design and findings of our review. We removed the lowest score on the ‘Measure of IQ’ scale, as in order to be included in the review studies had to report that all or part of their sample had a full scale IQ ≥ 70. We also removed the scale which in our review would be labelled as psychometric properties of the ADHD assessment measure, when used specifically with young people with ASD without ID. This is because none of the included studies provided data on this and so all studies would have scored at floor on this scale.

| **Scale** | **Criteria** | **Risk of bias score** |
| --- | --- | --- |
| **Diagnosis of ASD** | A clinical diagnosis was given by a psychologist or psychiatrist according to DSM-IV, DSM-V OR ICD-10 criteria | 2 (low risk) |
|  | A research diagnosis of ASD was made using a validated research measure (ADOS/ADI-R) by a researcher trained in its administration. | 1 (medium risk) |
|  | A diagnosis of ASD was made by a researcher using a checklist of DSM-IV, DSM-V or ICD-10 criteria | 0 (high risk) |
| **Assessment of ADHD symptoms** | A clinical diagnosis of ADHD was given by a psychologist or psychiatrist according to DSM-IV, DSM-V or ICD-10 criteria. Diagnosis based on both parent and teacher assessment. | 2 (low risk) |
|  | Use of validated research measure for assessment of ADHD (e.g. Conners-3 Parent Assessment). | 1 (medium risk) |
|  | Use of research measure that is not validated (e.g. tool that author has created to assess inattention and hyperactivity-impulsivity). | 0 (high risk) |
| **Clear description of participants** | Key characteristics described including: mean age, age range, gender, ethnicity, socioeconomic status | 2 (low risk) |
|  | Some descriptive characteristics included; or reference to whether further details can be found is provided | 1 (medium risk) |
|  | Key demographic information missing | 0 (high risk) |
| **Description of recruitment pool provided** | Recruitment pool described including: method of referral (e.g. self, database) and setting (e.g. clinic or school). | 2 (low risk) |
|  | Some detail provided | 1 (medium risk) |
|  | Recruitment pool is not described | 0 (high risk) |
| **Measure**  **of IQ** | IQ measured during study using standardised assessment tool | 2 (low risk) |
|  | All participants described as having an IQ > 70, no further details given | 1 (medium risk) |
| **Total score = X/10**  **0-3 = high risk of bias**  **4-7 = medium risk of bias**  **8-10 = low risk of bias** | | |

**References**

Hoy, D., Brooks, P., Woolf, A., Blyth, F., March, L., Bain, C., . . . Buchbinder, R. (2012). Assessing risk of bias in prevalence studies: modification of an existing tool and evidence of interrater agreement. *Journal of clinical epidemiology, 65*(9), 934-939.

Munn, Z., Moola, S., Riitano, D., & Lisy, K. (2014). The development of a critical appraisal tool for use in systematic reviews addressing questions of prevalence. *International journal of health policy and management, 3*(3), 123.

Wigham, S., Barton, S., Parr, J. R., & Rodgers, J. (2017). A systematic review of the rates of depression in children and adults with high-functioning autism spectrum disorder. *Journal of Mental Health Research in Intellectual Disabilities, 10*(4), 267-287.
